# Highly informed, yet undersupported: dietary supplement use, information sources, and perceived risks among pregnant vegans in German-speaking regions—findings from the VedieS study

**DOI:** 10.3389/fnut.2026.1866907

**Published:** 2026-07-21

**Authors:** Wolfgang Huber-Schneider, Karl-Heinz Wagner, Ingrid Kiefer

**Affiliations:** 1 Department of Nutritional Sciences, University of Vienna, Vienna, Austria; 2 Austrian Agency for Health and Food Safety GmbH, Vienna, Austria

**Keywords:** dietary supplements, information sources, micronutrients, nutrition counseling, plant-based nutrition, pregnancy, public health nutrition, vegan diet

## Abstract

**Background:**

Vegan diets during pregnancy are commonly discussed regarding nutritional risks, often without differentiating between heterogeneous vegan populations. Empirical evidence on clearly defined subgroups who maintain a vegan diet throughout pregnancy remains scarce.

**Objectives:**

This study characterized a highly selected subgroup of pregnant women adhering to a vegan diet, focusing on supplement use, information sources, counseling experiences, and perceived risks of micronutrient deficiency and overdosing.

**Methods:**

An explorative, cross-sectional online survey was conducted as part of the Vegan Diet and Dietary Supplements Study (VedieS). The questionnaire was developed from a scientific literature review, pilot-tested, and distributed via vegan, nutrition-related, and health-focused organizations and social media across German-speaking regions. Individuals adhering to a vegan diet (defined as a strictly plant-based diet excluding all animal-derived products) participated. The analysis was restricted to those reporting a vegan diet during at least one pregnancy. Supplement use, information seeking, and counseling adequacy were analyzed descriptively.

**Results:**

In total, 801 individuals participated. Participants were highly educated and predominantly from urban areas (76.3% tertiary education; 79.6% urban). Adherence during pregnancy was largely consistent (90.5%), and supplement use was nearly universal (99.1%) with high compliance (79.1%), targeting micronutrients critical in vegan pregnancies, including vitamin B12 (77.8%), omega-3 fatty acids (76.9%), folic acid (73.8%), and vitamin D (67.8%). Scientific literature, self-identified by participants, was the primary information source (80.3%), whereas professional counseling was less frequent and often perceived as insufficient. Although 74.7% informed their gynecologist, only 16.1% reported comprehensive counseling. Despite high motivation and self-directed information seeking, considerable uncertainty persisted regarding micronutrient dosage, particularly overdosing, with 40.4% perceiving dosage guidance as insufficient. External influences, including the social environment (46.3%) and physicians (34.2%), were relevant sources of uncertainty.

**Conclusion:**

Rather than a uniformly high-risk population, pregnant individuals following a vegan diet represent a highly informed and responsible subgroup whose main challenges relate less to dietary behavior than to structural gaps in professional counseling and access to consistent, evidence-based supplementation guidance. These findings underline the public health relevance of expanding nutrition training and counseling capacities among healthcare professionals and the need for clinical research using objective measures of nutritional status in vegan pregnancies.

## 1 Introduction

In recent years, vegan diets have gained substantial popularity across many Western countries, thereby increasing their relevance in nutritionally sensitive life stages such as pregnancy (1). A vegan diet is defined as a dietary pattern that excludes all animal-derived products - including meat, fish, dairy, eggs, and honey - and is based on the consumption of plant-derived foods such as fruits, vegetables, grains, legumes, nuts, and seeds (2). In German-speaking countries, recent estimates of the prevalence of a vegan diet range from approximately 1–2% of the population in Germany (3, 4) to around 5% in Austria (5) and 0.5–3% in Switzerland (6). Pregnancy is accompanied by profound physiological adaptations and a rise in the demand for both macro- and micronutrients, rendering an adequate and well-balanced nutrient supply essential for maternal health and optimal fetal development (7). A vegan diet can meet the nutritional requirements of a pregnancy when it is carefully planned and complemented by appropriate supplementation (8). Nevertheless, its safe implementation presupposes a high level of nutritional literacy, intentional dietary management, and the targeted use of dietary supplements (1, 9). Within this context, several micronutrients are repeatedly identified as potentially critical during vegan pregnancy, including vitamin B12, iron, iodine, calcium, zinc, vitamin D, and long-chain omega-3 fatty acids, particularly DHA and EPA (9, 10). Among these, vitamin B12 supplementation is consistently emphasized across professional recommendations as indispensable for individuals following a vegan diet (10). Professional nutrition societies differ in their assessment of vegan diets during pregnancy. While the Academy of Nutrition and Dietetics (AND) considers a well-planned vegan diet to be appropriate even during nutritionally sensitive life stages such as pregnancy, other organizations are more cautious (7, 10). The German Nutrition Society emphasizes the limited evidence base and therefore does not issue a clear recommendation for or against a vegan diet for vulnerable groups such as pregnant women. It highlights the potential risk of adverse outcomes in cases of inadequate implementation and emphasizes the need for advanced nutritional competence and qualified, individualized counseling (11). In contrast, the Austrian Nutrition Society does not recommend a vegan diet during pregnancy due to concerns about possible malnutrition and associated health risks (12). Despite these differing positions, there is consensus that vegan diets during pregnancy require careful planning and particular attention to critical micronutrients, most notably vitamin B12, and that professional nutritional counseling is essential to ensure adequate nutrient supply. Evidence regarding pregnancy outcomes associated with vegan diets still remains inconsistent. Recent observational studies and systematic reviews have reported variations in birth outcomes, such as lower mean birth weight or a higher proportion of infants born small for gestational age, among some vegan or strictly vegetarian populations compared with omnivorous reference groups. However, these findings differ substantially across studies, derive from a still limited and partly small body of evidence, and appear to be strongly influenced by methodological approaches and population characteristics (13–15). Rather than supporting generalized assumptions about vegan diets as inherently risky, these observations underscore the central importance of adequate nutrient supply during pregnancy (9). Maintaining a vegan diet during pregnancy may require sustained dietary adherence and active nutritional self-management. However, empirical evidence regarding determinants of adherence to vegan diets during pregnancy remains limited. Existing studies in pregnant populations primarily focus on nutritional status, health outcomes, or information needs, and only a small number have explored experiences related to maintaining a vegan dietary pattern during pregnancy (1, 15–17). Qualitative evidence suggests that adherence may be influenced by ethical convictions, health-related motivations, social support, perceived stigma, pregnancy-related symptoms, and access to professional dietary guidance (16, 17). Evidence from non-pregnant vegan and vegetarian populations provides additional insights into factors associated with long-term dietary adherence. Studies have identified moral and ethical motivations, health beliefs, environmental concerns, nutritional knowledge, social support, and food availability as important facilitators of adherence, whereas social barriers, uncertainty regarding nutritional adequacy, and practical challenges in everyday life may hinder sustained dietary compliance (3, 18–21). Several studies further suggest that ethical and moral motivations may contribute to stronger long-term adherence to vegan dietary patterns than health-related motives alone (19, 20, 22). Although these findings cannot be directly extrapolated to pregnancy, they indicate that maintaining a vegan diet during this life stage is likely influenced by a complex interplay of individual, social, and healthcare-related factors. In addition to personal motivations, access to reliable information and professional counseling may be particularly relevant during pregnancy, when nutritional requirements increase and dietary decisions are closely linked to concerns about maternal and fetal health (7, 16, 17). Nevertheless, little is known about how pregnant individuals following a vegan diet obtain nutrition-related information, select dietary supplements, and navigate potentially conflicting recommendations from healthcare professionals, scientific sources, and online resources. Beyond physiological requirements, dietary practices during pregnancy are influenced by a range of influencing factors, including access to reliable information, availability and quality of professional counseling, and prevailing social attitudes toward vegan diets (7, 23). In many countries, dietary supplements are regulated as foods rather than pharmaceuticals, and recent investigations have demonstrated considerable variability in product composition, labeling accuracy, and overall quality of prenatal supplements (24–26). In this setting, inconsistent or ambiguous guidance regarding supplement selection and dosage may contribute to uncertainty, particularly among pregnant individuals (26). To date, research on vegan diets during pregnancy has frequently approached vegan populations in a generalized manner. Empirical evidence describing how individuals who maintain a vegan diet throughout pregnancy manage their nutritional intake, which information sources they prioritize, and how they evaluate potential risks related to micronutrient intake remains limited (1, 15). In particular, determinants of supplementation behavior and information-seeking practices within this subpopulation have not been sufficiently characterized (15, 17). A more differentiated understanding of these aspects is therefore required to contextualize a vegan diet during pregnancy and to identify potential gaps in professional counseling and public health strategies.

Against this background, the present cross-sectional study was conducted as part of the Vegan Diet and Dietary Supplements Study (VedieS), a broader explorative survey on vegan diets and dietary supplement use during nutritionally sensitive life stages, approved by the Ethics Committee of the University of Vienna (reference number 01021). It aimed to characterize pregnant individuals adhering to a vegan diet in German-speaking countries and regions regarding dietary supplement use, sources of information influencing supplementation decisions, and perceived risks of micronutrient under- and overdosing.

## 2 Materials and methods

### 2.1 Study design

The present study is based on an explorative, cross-sectional online survey. Analyses were restricted to participants who reported adherence to a vegan diet during at least one pregnancy.

### 2.2 Questionnaire development

The questionnaire was developed by the authors (Wolfgang Huber-Schneider, Karl-Heinz Wagner, Ingrid Kiefer) and was informed by an extensive review of the scientific literature on vegan diets and dietary supplement use during nutritionally sensitive life stages. It addressed several thematic domains, including sociodemographic characteristics; conceptions of health during pregnancy; vegan dietary patterns and motivations; knowledge, attitudes, and perceived benefits and risks of dietary supplements; supplement selection and use during and outside pregnancy; sources of information and counseling experiences; and perceived risks of micronutrient under- and overdosing. In total, the questionnaire comprised 55 items, of which two were open-ended (free-text) and 53 were closed-ended, using single-choice and multiple-choice questions, Likert-scale items, and ranking tasks. As an example, one item assessed the perceived adequacy of dosage guidance (“How do you evaluate the available advice/information on dosage of dietary supplements during pregnancy?”; response options: very good, good, adequate, inadequate, or “do not know”). A further item recorded the dietary supplements used during pregnancy (“Which dietary supplements did you take or are you taking during your pregnancy/pregnancies?”; multiple answers possible, with response options including vitamin B12, folic acid, omega-3 fatty acids (DHA/EPA), iron, vitamin D, iodine, zinc, calcium, magnesium, combination products, none, or other). An adaptive structure with filter and branching logic ensured that participants were presented only with questions relevant to their previous responses. Prior to launching the main survey, the questionnaire was pretested with 20 individuals adhering to a vegan diet to assess clarity, structure, and technical functionality, and minor adjustments were made based on this feedback before study initiation. The full questionnaire is provided as Supplementary material. For participants who had experienced more than one pregnancy, all pregnancy-related questions referred to their current or most recent pregnancy.

### 2.3 Survey distribution and recruitment

Recruitment was conducted between October 2023 and July 2025 via public announcements, newsletters, and social media platforms (e.g., Facebook, Instagram). Distribution of the survey was supported by more than 15 vegan, nutrition-related, and health-focused organizations across German-speaking countries and regions, including Germany, Austria, Switzerland, Luxembourg, Liechtenstein, and South Tyrol. Because the questionnaire was provided in German, participants needed sufficient knowledge of the language. Participation was voluntary and anonymous. Data were collected using an online questionnaire administered via LimeSurvey (LimeSurvey Cloud, Version 6.6.7). The questionnaire could be completed at any time and from any location using a smartphone, tablet, or computer, with an estimated completion time of 20–30 min. All questions displayed to participants were mandatory in order to complete the questionnaire. Consequently, no missing data occurred within the respective question blocks. Percentage values are therefore reported as valid percentages relative to the relevant subgroup of participants who were presented with and completed a given item.

### 2.4 Inclusion and exclusion criteria

Participants were eligible for inclusion if they (1) were at least 18 years of age, (2) reported adherence to a vegan diet during at least one pregnancy, and (3) provided digital informed consent after reviewing the participant information. For the purpose of this study, a vegan diet was defined as a strictly plant-based diet excluding all animal-derived products (i.e., meat, fish and seafood, dairy products, eggs, and honey or other bee products); this definition was operationalized through the dietary self-classification item of the questionnaire, so that participants assessed their own dietary pattern against it. Participants who reported more than two vegetarian or omnivorous dietary exceptions per month (independent of pregnancy) were excluded from the analysis, whereas rare exceptions (≤2 times per month) did not result in exclusion, in order to reflect real-world dietary practices more accurately. After applying these criteria, 801 individuals adhering to a vegan diet were retained in the survey sample (Table 1); the present analysis was restricted to women and other/non-binary participants aged ≥18 years who reported following a vegan diet during a current or previous pregnancy (*n* = 757).

**Table 1:** Characteristics of the study population (*n* = 801).

Characteristic	Value %
Sex	
Female	94.0
Male	5.5
Diverse/Other/Non-binary	0.5
Age (years)	
21–30	22.5
31–40	67.9
41–50	8.1
51–60	1.5
Country of residence	
Austria	27.7
Germany	63.7
Switzerland	6.4
Other countries	2.2
Area of residence	
Urban (>10.000 inhabitants)	79.6
Rural (≤10.000 inhabitants)	20.4
Education level	
Tertiary education^a^. %	76.3
Household net income (monthly EUR/CHF)	
≤2.000	5.6
2.001–4.000	40.5
≥4.001	49.4
Duration of vegan diet	
1–3 years	12.5
3–5 years	29.0
5–10 years	36.7
>10 years	21.0
Other durations (<1%)	0.8
Parent of at least one child	86.0
Currently pregnant	22.3

a Tertiary education includes university and equivalent higher education.

### 2.5 Data analysis

Statistical analyses were conducted using IBM SPSS Statistics (Version 29; IBM Corp., Armonk, NY, United States). The software was used for descriptive statistical analyses as well as for the generation of tables and figures. Given the exploratory nature of the study and the predominantly categorical and ordinal structure of the data, analyses were primarily descriptive. Categorical variables are presented as absolute frequencies and percentages, while ordinal variables derived from Likert-scale items are reported as medians with interquartile ranges (IQR). For selected variables, response patterns were additionally examined descriptively across sociodemographic characteristics, including age group, educational level, and place of residence (urban vs. rural). These exploratory comparisons were intended to illustrate response patterns and did not involve formal statistical testing or hypothesis testing. Visualizations were created to illustrate supplementation practices, perceived risks, and information-related uncertainties across micronutrients and participant subgroups. All figures were generated directly using SPSS.

### 2.6 Ethical considerations

The study was approved by the Ethics Committee of the University of Vienna (reference number: 01021). Before starting the survey, all participants received detailed information regarding study objectives, procedures, duration, and data protection, and provided digital informed consent.

## 3 Results

### 3.1 Study population and sociodemographic characteristics

In total, 801 individuals reporting general adherence to a vegan diet participated in the online survey. The study population consisted predominantly of women (94.0%), with smaller proportions of male (5.5%) and other/non-binary (0.5%) participants, and was mainly recruited from German-speaking countries, with the largest proportions residing in Germany (63.7%), Austria (27.7%), and Switzerland (6.4%), while 2.2% reported residence in other countries (Table 1). Participants were primarily aged 31–40 years (67.9%), followed by 21–30 years (22.5%), 41–50 years (8.1%), and 51–60 years (1.5%). At the time of participation, 22.3% of respondents reported being currently pregnant. Overall, participants were characterized by a high level of educational attainment, with 76.3% reporting tertiary education, predominantly urban residence (79.6%), and comparatively high socioeconomic status, with 49.4% reporting a household net income ≥ 4.001 (EUR/CHF) per month, 40.5% between 2.001–4.000, and 5.6% ≤ 2.000 (Table 1). Most participants had followed a vegan diet for several years, with 36.7% reporting 5–10 years, 29.0% 3–5 years, 12.5% 1–3 years, and 21.0% more than 10 years of adherence.

### 3.2 Timing of adoption of a vegan diet and dietary patterns during pregnancy

The vast majority of participants had adopted a vegan diet prior to pregnancy (86.0%), whereas only a very small proportion initiated veganism during (2.3%) or between pregnancies (11.7%) (Table 2). Accordingly, adherence to a vegan diet during pregnancy predominantly reflected long-lasting dietary practice rather than a temporary or situational change. The small group who reported adopting a vegan diet only during pregnancy (2.3%) nevertheless followed a vegan diet throughout the relevant pregnancy and therefore met the inclusion criterion. Dietary patterns during the current or most recent pregnancy were largely stable. More than 90% of participants (90.5%) reported continuing a vegan diet throughout pregnancy, while only a small minority temporarily shifted to a vegetarian diet (6.5%) or reported other deviations, including consumption of fish or meat (3.0%) (Table 2); such exceptions remained rare (≤ two per month) and therefore did not affect eligibility. Independent of pregnancy status, 63.4% of participants described themselves as strictly vegan, while a relevant proportion reported rare vegetarian exceptions, including occasional consumption of honey or other beekeeping products (16.7%) or dairy products (17.1%).

**Table 2:** Attitudes, perceptions, and experiences regarding dietary supplements during and outside vegan pregnancy.

Item	Response	Participants %
General adherence to a vegan diet (independent of pregnancy)	Always strictly vegan	63.4
	Almost always vegan, rare use of honey and/or beekeeping products	16.7
	Almost always vegan, rare use of dairy products	17.1
Frequency of deviations from a vegan diet (independent of pregnancy)	Once or twice per year	15.3
	Once or twice per half-year	37.4
	Once or twice per month	32.1
	Once or twice per week	15.3
Timing of adoption of a vegan diet	Before pregnancy/pregnancies	86
	During pregnancy	2.3
	Between pregnancies	11.7
Dietary pattern during the most recent/current pregnancy	Continued vegan diet	90.5
	Vegetarian diet	6.5
	Consumption of fish and/or meat	3
Dietary supplement use and compliance	Use of dietary supplements during pregnancy (yes)	99.1
	Always used supplements during pregnancy	79.1
	Mostly used supplements during pregnancy	16.3
Vegan-specific aspects	Pays attention to supplements being vegan (yes)	97.8
	Informed gynecologist about vegan diet during pregnancy (yes)	74.7
What does “health during pregnancy” primarily mean to you?	Complication-free pregnancy	29.3
	Fetal development consistent with medical standards	11.7
	Optimal fetal development through adequate nutrient supply	45.9
How are dietary supplements perceived?	Comparable to over-the-counter medicines	18.5
	Comparable to foods	34.3
	Not comparable to foods or medicines	42.3
Perceived health effects of dietary supplements	Dietary supplements can positively affect health	94.3
	Supplement use is particularly beneficial for vegans	88.5
Dietary supplement use and compliance	Use of dietary supplements during pregnancy (yes)	99.1
	Always used supplements during pregnancy	79.1
	Mostly used supplements during pregnancy	16.3
Information and counseling	Previously received counseling on dietary supplements (yes)	60.5
	Desire for more counseling by physicians, dietitians, or pharmacists (yes)	97.5
	Information on supplements during pregnancy is easily accessible (yes)	52.1
	Information on supplements during pregnancy is easily accessible (no)	44.2
Perceived adequacy of counseling on supplement dosage	Not sufficient	40.4
	Sufficient	29.6
	Good to very good	28
Self-perceived knowledge and attitudes	Feels comprehensively informed about supplements during pregnancy (yes)	92.9
	Believes nutrient deficiencies can be compensated by supplements (yes)	95.9
Counseling by gynecologist during pregnancy	Comprehensive counseling	16.1
	Hardly counseled	59.1
	Not counseled at all	13
	Did not wish to be counseled	5.6
Main concerns regarding supplement use	Potential harm to the unborn child due to incorrect dosage	35.5
	No concerns, supplements are considered safe	42.8

Percentages may not sum to 100% due to rounding or exclusion of minor categories. The items on general adherence to a vegan diet and on the frequency of deviations (both assessed independently of pregnancy) constitute the screening stage used to define the survey sample: the general-adherence item was answered by all respondents (*n* = 846) and the deviation-frequency item only by those reporting occasional deviations; participants reporting more than two vegetarian or omnivorous exceptions per month (*n* = 45) were excluded, yielding the survey sample (*n* = 801). All pregnancy-specific items refer to the analysis subgroup (*n* = 757; see Section 2.4).

### 3.3 Perceptions of health and attitudes toward dietary supplements

Health during pregnancy was primarily conceptualized in terms of optimal fetal development, with particular emphasis on adequate nutrient supply (45.9%), followed by a complication-free pregnancy (29.3%) and fetal development consistent with medical standards (11.7%) (Table 2). Dietary supplements were regarded as highly relevant to health by nearly all participants, and 99.1% reported using dietary supplements during pregnancy. A large majority perceived dietary supplements as beneficial, especially for individuals following a vegan diet (88.5%), and 94.3% reported that dietary supplements can positively affect health. At the same time, participants demonstrated a differentiated understanding of dietary supplements, which were predominantly viewed as a category distinct from both foods and medications (42.3%), while others considered them comparable to foods (34.3%) or over-the-counter medicines (18.5%) (Table 2). Participants’ personal understanding of what constitutes a dietary supplement was captured through a dedicated questionnaire item (“What are dietary supplements?”); no standardized definition was presented beforehand, and the response options reflected standard legal and scientific definitional components (Supplementary material).

### 3.4 Dietary supplement use during vegan pregnancy

**Figure 1:**
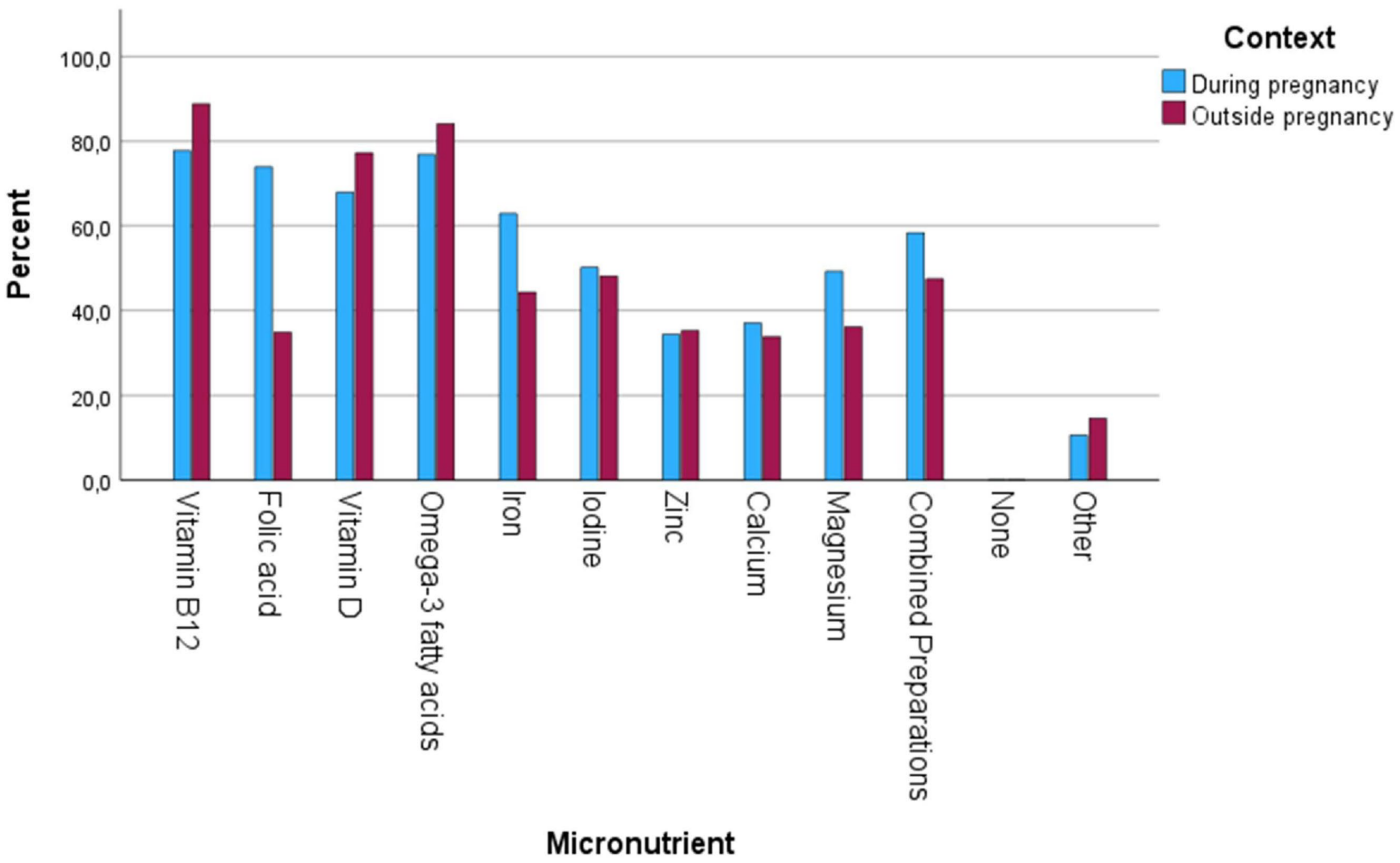
Dietary supplement use during and outside pregnancy among vegans.

Dietary supplement use during pregnancy was nearly universal and marked by high compliance. Most participants (79.1%) reported consistent supplement intake throughout pregnancy (Table 2). Supplementation during pregnancy primarily included micronutrients commonly regarded as critical for vegans in general and in vegan pregnancies, including vitamin B12 (77.8%), vitamin D (67.8%), omega-3 fatty acids (76.9%), iodine (50.1%), iron (62.8%), and folic acid (73.8%) (Table 3). General supplementation patterns among vegans outside pregnancy were largely comparable to those observed during vegan pregnancy, with the notable exception of folic acid, which was supplemented less frequently outside pregnancy (Table 3; Figure 1). Almost all participants indicated that they paid close attention to the vegan status of consumed supplements (97.8%), reflecting the consistent application of dietary principles during a nutritionally sensitive life stage.

**Table 3:** Use of dietary supplements during and outside pregnancy among vegans.

Micronutrient	During pregnancy (%)	Outside pregnancy (%)
Vitamin B12	77.8	88.8
Folic acid	73.8	34.8
Vitamin D	67.8	77.3
Omega-3 fatty acids	76.9	84.1
Iron	62.8	44.3
Iodine	50.1	48.1
Zinc	34.3	35.2
Calcium	37.0	33.8
Magnesium	49.2	36.1
Combined preparations	58.3	47.4
None	0.2	0.2
Other	10.6	14.6

Multiple responses were allowed. Participants could report the use of more than one supplement. Combined preparations refer to combination products such as (prenatal) multivitamins. The category “Other” comprises any supplement not listed above and was not further specified by participants. “During pregnancy” refers to supplement use during pregnancy; “outside pregnancy” refers to vegans not currently pregnant.

### 3.5 Information sources, counseling, and external influences

Information seeking related to a vegan diet and supplementation during pregnancy was largely self-directed. Scientific literature, as self-identified by participants, emerged as the most frequently reported information source (80.3%), followed by internet and social media (47.2%), vegan organizations (46.7%), other vegans (35.1%), and gynecologists (37.2%), whereas medical professionals were less commonly cited as primary sources of information, including dietitians (17.2%) and general practitioners (14.9%) (Table 4).

**Table 4:** Sources of information used for micronutrient supplementation decisions during pregnancy.

Source of information	% reporting use
Social environment	5.2
General practitioner	14.9
Pediatrician	9.0
Gynecologist	37.2
Pharmacist	3.2
Dietitian	17.2
Midwife	17.7
Other vegans	35.1
Vegan organizations	46.7
Scientific literature	80.3
Internet and social media	47.2

Multiple responses were allowed. Percentages do not sum to 100%.

A notable discrepancy was observed between the information sources most frequently used and those perceived as providing optimal advice. While scientific literature remained the primary reference (37.5%), dietitians (18.7%) were rated more favorably than internet-based sources (2.7%) when optimal advice was considered (Table 5). External influences nevertheless played a relevant role. The most frequently reported sources of uncertainty were the social environment (46.3%), physicians (34.2%), and a lack of trustworthy and consistent information (27.8%) (Table 6). Although approximately three quarters of participants reported informing their gynecologist about their vegan diet (74.7%), only a minority described having received comprehensive counseling on dietary supplement use (16.1%), while 59.1% reported receiving little counseling and 13.0% none. At the same time, nearly all participants expressed a strong desire for increased professional support from physicians, dietitians, and pharmacists (97.5%) (Table 2).

**Table 5:** Sources perceived as providing the best advice on micronutrient supplementation during pregnancy.

Source of information	% reporting use
General practitioner	2.0
Pediatrician	0.4
Gynecologist	10.6
Pharmacist	0.7
Dietitian	18.7
Midwife	2.7
Other vegans	5.4
Vegan organizations	15.5
Scientific literature	37.5
Internet and social media	2.7
Total	100.0

Single response only. Medical professionals are reported separately due to differing professional roles and expertise.

**Table 6:** Factors perceived as discouraging a vegan diet during pregnancy.

Discouraging factor	% reporting influence
Social environment	46.3
Physicians	34.2
Pharmacists	2.4
Dietitians	0.6
Midwife	4.4
Internet and social media	13.2
Lack of trustworthy information	27.8
Not discouraged	25.7

Multiple responses were allowed. Percentages do not sum to 100%.

### 3.6 Perceived information gaps and uncertainties regarding dosage

**Figure 2:**
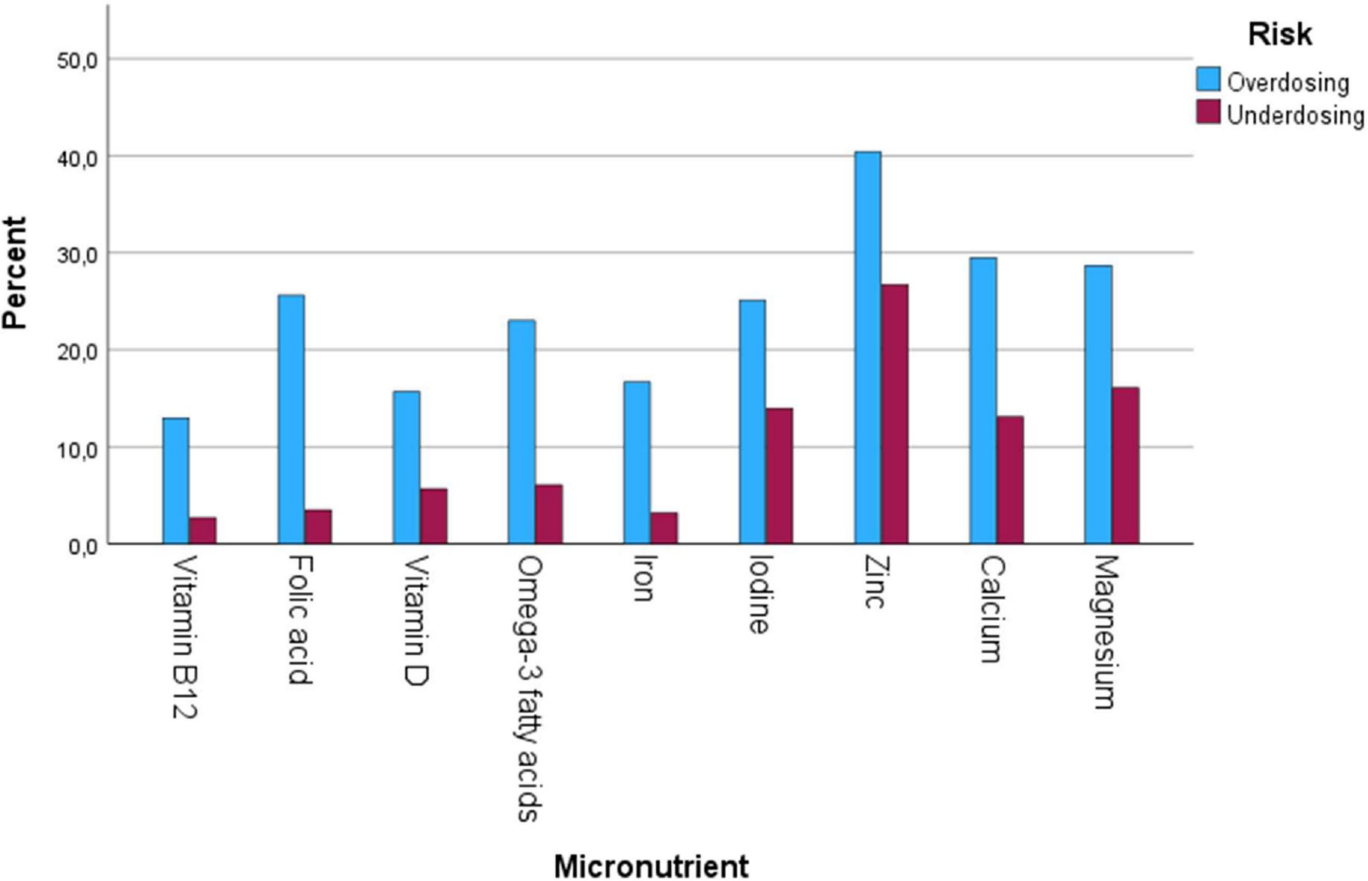
“Do not know” responses regarding perceived risks of micronutrient under- and overdosing during pregnancy.

A substantial proportion of participants reported difficulties in accessing reliable information on dietary supplements during pregnancy, with 44.2% indicating that information access was challenging, while 52.1% considered information to be easily accessible (Table 2). In addition, approximately 40.4% perceived the available guidance on supplement dosage as insufficient, whereas 29.6% considered it sufficient and 28.0% rated it as good or very good (Table 2). These perceived gaps were also reflected in risk assessments. While risks related to micronutrient deficiency during pregnancy were generally acknowledged, uncertainty regarding potential risks of overdosing was recognizable. For several micronutrients, a considerable number of participants selected “do not know,” including 40.4% for zinc, 29.5% for calcium, 28.7% for magnesium, 25.6% for folic acid, 25.1% for iodine, and 23.0% for omega-3 fatty acids, indicating persistent knowledge gaps even within this highly informed and motivated vegan population subgroup (Table 7; Figure 2).

**Table 7:** Perceived risks of micronutrient under- and overdosing during pregnancy among vegans.

Micronutrient	Underdosing median (IQR)	% “do not know”	Overdosing median (IQR)	% “do not know
Vitamin B12	4 (4–5)	2.7	2 (1–3)	13.0
Folic acid	5 (4–5)	3.5	2 (1–3)	25.6
Vitamin D	4 (3–5)	5.7	3 (2–4)	15.7
Omega-3 fatty acids	4 (3–5)	6.1	2 (1–3)	23.0
Iron	4 (4–5)	3.2	3 (2–4)	16.7
Iodine	4 (3–5)	14.0	4 (3–4)	25.1
Zinc	3 (3–4)	26.7	3 (2–3)	40.4
Calcium	4 (3–4)	13.1	3 (2–3)	29.5
Magnesium	3 (3–4)	16.1	2 (1–3)	28.7

Risk perception was assessed on a 5-point Likert scale (1 = very low risk, 5 = very high risk). “Do not know” responses were excluded from median and interquartile range calculations. All remaining responses contributed to the 1–5 ratings.

## 4 Discussion

This study provides a rare and detailed look at a clearly defined subgroup within the vegan population: vegans who maintain a vegan diet throughout pregnancy. Across the descriptive subgroup comparisons, response patterns regarding supplementation, information sources, and perceived nutritional risks appeared broadly similar across age groups, educational levels, and places of residence. As these comparisons were exploratory and descriptive rather than based on formal statistical testing, they should be interpreted with caution. These observations are nonetheless consistent with the interpretation that pregnant vegans in this sample may represent a relatively coherent subgroup rather than a loosely connected or experimentally motivated dietary collective, although this descriptive interpretation should be regarded as tentative given the self-selected nature of the sample. The age distribution may be consistent with this interpretation, as a substantial proportion of participants (67.9%) were between 31 and 40 years of age. It is possible that this age range coincides with more established life circumstances; however, the present data do not allow conclusions about whether such factors account for the high levels of nutritional engagement, information seeking, and consistent supplementation observed in this cohort. Previous research has often addressed vegan or plant-based diets as a single category, which may hide meaningful differences in nutritional competence, motivation, and experience (13–15, 27, 28). At the same time, inconsistencies in nutritional counseling for plant-based diets during pregnancy have been documented, with several studies indicating variable knowledge levels and limited confidence among healthcare professionals, often attributed to insufficient nutrition training (18, 29, 30).

Hence, the present findings are consistent with the interpretation that individuals who adhere to a vegan diet during pregnancy and thereafter form a particularly selected subgroup within the broader vegan population. Within this sample, participants clearly did not conform to persistent stereotypes portraying vegans as rigid, ideologically driven, or nutritionally negligent, but instead demonstrated a reflective and pragmatic dietary orientation. Although the voluntary nature of participation likely attracted particularly engaged and motivated individuals, the data nonetheless offer meaningful evidence that vegan pregnancy can be approached in a well-informed and considered way, even if this pattern may be less pronounced in less engaged segments of the vegan population.

Strong adherence to vegan principles coexisted with rare and situational dietary exceptions, suggesting a flexible and pragmatic approach to maintaining a vegan diet during pregnancy. This pattern is also reflected in the stability of dietary practices observed in the present cohort, where more than 90% of participants reported maintaining a vegan diet throughout pregnancy and 63.4% described themselves as strictly vegan. These observations align with emerging qualitative evidence indicating that vegan pregnancy is often characterized by a combination of confidence, responsibility, and adaptability, alongside exposure to social skepticism and limited professional support (15, 16).

Within this sample, the near-universal use of dietary supplements is difficult to reconcile with assumptions that vegan pregnancies are inherently associated with careless nutrient management (15, 31). In the present study, 99.1% of participants reported using dietary supplements during pregnancy, with 79.1% indicating consistent supplementation throughout this life stage. Recent reviews emphasize that plant-based diets can adequately support pregnancy when appropriately supplemented and when professional guidance is available, particularly through dietetic expertise (8, 32). Within the present subgroup, concerns about systematic neglect of supplementation therefore appear largely unfounded; however, given the high educational attainment, socioeconomic status, and engagement that characterized this self-selected sample, this pattern may not extend to less informed or less resourced individuals adhering to a vegan diet during pregnancy.

At the same time, the findings reveal a persistent and clinically relevant uncertainty regarding micronutrient dosage, especially in relation to potential overdosing (15, 17, 33). This observation is notable given the high levels of self-perceived knowledge and engagement reported by participants. Similar concerns have been raised in recent expert consensus statements, which highlight inconsistencies across guidelines and a lack of clear, practice-oriented dosage recommendations for pregnancy (7, 29). The absence of unified, accessible guidance may therefore contribute to uncertainty even among highly informed individuals.

An additional aspect that warrants consideration is the potential impact of nutritional deficiencies and the challenges associated with their assessment during pregnancy. Adequate supplementation is particularly important for nutrients that are frequently identified as critical during pregnancy, including vitamin B12, iron, iodine, vitamin D, calcium, zinc, and long-chain omega-3 fatty acids. Deficiencies in these nutrients have been associated with adverse maternal and fetal outcomes, whereas appropriate supplementation may contribute to improved nutritional status and pregnancy outcomes (14, 15, 31, 34, 35). This issue may be particularly relevant for individuals following restrictive dietary patterns such as vegan diets, where nutritional adequacy depends on careful dietary planning and targeted supplementation (14, 15, 31).

At the same time, the biochemical assessment of micronutrient status during pregnancy is inherently complex. Physiological adaptations, including plasma volume expansion, altered protein concentrations, and changes in nutrient metabolism, can substantially influence circulating biomarker levels and complicate their interpretation (36, 37). Furthermore, the selection of biomarkers, analytical methods, and assay performance may affect the accuracy and comparability of laboratory measurements, and pregnancy-specific reference ranges are not consistently available for all micronutrients (36–38). Consequently, both nutritional counseling and laboratory assessment should be interpreted within the broader physiological context of pregnancy, highlighting the importance of individualized evaluation and evidence-based guidance.

A central finding of this study is the discrepancy between the strong demand for professional counseling and the limited counseling actually received, with 97.5% of participants expressing a desire for increased professional support, while only 16.1% reported having received comprehensive counseling. Although most participants reported informing their gynecologist about their vegan diet, comprehensive advice on dietary supplements was uncommon. Existing literature suggests that healthcare professionals’ willingness and confidence to provide nutrition counseling are influenced by their own training, beliefs, and familiarity with plant-based diets (39). While some obstetricians and gynecologists regard well-planned vegan diets as safe, others report uncertainty or skepticism rooted in misconceptions or limited nutritional education (18, 30).

In the absence of consistent professional guidance, responsibility for nutritional decision-making increasingly shifts to pregnant individuals themselves. Scientific literature (80.3%), vegan organizations (46.7%), and internet or social media (47.2%) therefore emerged as important sources of information. The category “scientific literature,” self-identified by participants, may include a wide range of materials, from peer-reviewed scientific publications to popular science or health-related publications, informational resources addressing medical or nutritional topics, or educational materials commonly available in healthcare settings such as medical offices or waiting rooms. The exact scientific quality of the consulted sources could therefore not be assessed. While such self-directed information seeking may be feasible for a highly educated and motivated subgroup, it entails inherent risks given the variability in quality, relevance, and interpretability of available information. These findings underscore the structural gap between existing nutritional knowledge and its consistent implementation in everyday healthcare settings (17, 18, 29, 40).

This highly informed subgroup of vegan pregnant women may serve as a valuable reference point for identifying real-world challenges of optimally planned vegan diets during pregnancy. If uncertainties persist even under conditions of high motivation, compliance, and engagement, they are likely to be amplified among less experienced or less well-informed vegan individuals. These findings highlight the need to strengthen evidence-based professional counseling for vegan pregnancies. Rather than marginalizing or discouraging vegan diets during pregnancy, healthcare systems may benefit from strengthening provider training and expanding access to evidence-based nutrition counseling. This recommendation is supported by evidence suggesting that nutrition counseling interventions can improve dietary behaviors during pregnancy (41), whereas insufficient provider knowledge and confidence remain barriers to the consistent delivery of evidence-based dietary guidance, particularly in the context of plant-based nutrition (18). For instance, practice-oriented guidance specifically addressing vegetarian and vegan nutrition around pregnancy has been issued by professional bodies such as the Royal Dutch Organization of Midwives (KNOV) (42).

Pregnant women following a vegan diet should not be viewed as a vulnerable or irresponsible group. Instead, they represent a resilient, reflective, and health-conscious population whose primary challenge lies not in dietary behavior itself, but in insufficient professional support. While the findings cannot be generalized to all individuals following vegan diets, they provide important insights into information needs and structural gaps of a vulnerable group with clear relevance for public health nutrition.

Given the increasing prevalence of vegan diets in Western societies, strengthening evidence-based counseling for vegan pregnancies represents an emerging priority for public health nutrition. Future research should therefore complement survey-based insights with clinical investigations incorporating objective measures such as biomarkers, blood analyses, and standardized assessments of nutritional status. The highly motivated and nutritionally engaged subgroup identified in the VedieS study may represent a particularly suitable population for such investigations, as their sustained dietary patterns, high supplementation compliance, and strong health awareness allow the assessment of nutritional status under well-planned vegan dietary conditions. In this context, such populations may provide a more informative reference for evaluating potential risks of vegan diets than heterogeneous vegan samples that include individuals with widely differing levels of nutritional knowledge, motivation, and dietary adherence.

### 4.1 Summary of key findings

Overall, the self-reported data indicate that the investigated population represents a highly selected, experienced, and well-informed subgroup within the vegan population. This subgroup is characterized by strong adherence to vegan dietary practices, a high willingness to use dietary supplements, pronounced self-directed information seeking, and resilience toward external influences. Nevertheless, relevant uncertainties persist, particularly with respect to micronutrient dosage and access to consistent, evidence-based professional counseling.

### 4.2 Strengths and limitations

The present study provides detailed insights into a clearly defined and rarely investigated subgroup within the vegan population: individuals who maintain a vegan diet during pregnancy. By focusing specifically on this population, the analysis offers a differentiated perspective on supplementation practices, information-seeking behavior, and perceived counseling needs in a group that is frequently discussed but seldom examined as a distinct population. The relatively large sample size and the structured questionnaire enabled a systematic assessment of attitudes, information sources, and perceived nutritional risks. At the same time, the selective nature of the study population allows focused insights into nutritional decision-making and information acquisition within a subgroup that is particularly exposed to social, medical, and informational scrutiny.

Despite these strengths, several boundary conditions frame the interpretation of the findings. As an explorative, cross-sectional survey based on self-reported data without biomarker-based assessment, it cannot establish causal relationships or verify nutritional status. By design, the study focuses on individuals who maintain a vegan diet throughout pregnancy rather than those who discontinue it; the high adherence observed accordingly reflects this defined scope rather than pregnant vegans in general, and discontinuation lies outside its aim. As participation was voluntary, the findings describe a highly motivated, well-informed group and are not intended to represent the wider vegan population. Because some questions referred to earlier pregnancies and the exact time since each was not recorded, recall bias cannot be excluded; as recall declines with elapsed time, it likely affects mainly the older minority (41–50, *n* = 65; 51–60, *n* = 12), whereas the majority, aged 40 or younger (21–30, *n* = 180; 31–40, *n* = 544; total *n* = 801), reported on more recent pregnancies. The counseling context and availability of vegan products may also have changed over time. Conversely, the 22.3% pregnant at the time of the survey had not yet completed a full pregnancy and could not report on all of its challenges, but, being currently pregnant, provided the most current recollections. Restricting the analysis to recent pregnancies was deliberately avoided, as this hard-to-reach group would otherwise have yielded too small a sample; recruitment across several German-speaking countries (Table 1) with broadly similar healthcare standards makes country-level differences likely minor.

## 5 Conclusion

The present study demonstrates that pregnant individuals following a vegan diet constitute a highly informed, reflective, and health-conscious subgroup within the broader vegan population. This group is characterized by strong adherence to supplementation practices, high compliance, and pronounced engagement in self-directed information seeking. Despite this high level of motivation and nutritional awareness, even well-informed individuals encounter clear limits, most notably due to the absence of consistent, practice-oriented, and professionally delivered guidance on dietary supplementation and micronutrient dosage during pregnancy.

These findings suggest that the main challenge associated with vegan pregnancies may lie less in dietary behavior itself than in structural gaps in professional counseling and accessible expertise. Rather than categorizing vegans as a homogeneous risk group for nutrient deficiencies, healthcare systems should recognize the substantial heterogeneity within vegan populations and improve the availability of competent and accessible counseling across healthcare settings, including both specialized and low-threshold services.

As vegan diets continue to gain relevance in Western societies, ensuring that competent nutritional guidance becomes as accessible as the information sought by these individuals will represent a key challenge for future public health nutrition.

## Data Availability

The datasets presented in this article are based on anonymized survey data. Due to ethical considerations and the potential risk of indirect identification, the full dataset is not publicly available. Data may be made available upon reasonable request to the corresponding author.
