# Supplementary material for: Highly informed, yet undersupported: dietary supplement use, information sources, and perceived risks among pregnant vegans in German-speaking regions—findings from the VedieS study
Source: Front Nutr. 2026 Jul 21;13:1866907. doi: 10.3389/fnut.2026.1866907 (PMC13433308; doi:10.3389/fnut.2026.1866907)
Supplement: Supplementary file 1 [file supplementary_file_1.pdf]

General/Demographics

Gender *

Please select only one of the following answers:

- Female
- Male
- Diverse/Other/Non-binary

Age (in years) *

Please select only one of the following answers:

- Under 18
- 18-20
- 21-30
- 31-40
- 41-50
- 51-60
- 61 or older

Highest Completed Education *

Please select only one of the following answers:

- Compulsory School
- Apprenticeship/Vocational Training
- High School Diploma (Matura/Abitur)
- College
- University of Applied Sciences
- University

• Other

Monthly Net Household Income (including all household members, subsidies, and benefits) - in EUR or CHF *

Please select only one of the following answers:

• Up to 1,500

• 1,501 - 2,000

• 2,001 - 2,500

• 2,501 - 3,000

• 3,001 - 3,500

• 3,501 - 4,000

• Over 4,001

• Prefer not to say

Household Size (including children) *

Please select only one of the following answers:

• 1

• 2

• 3

• 4 or more

In which country do you live? *

Please select only one of the following answers:

• Austria

• Germany

• Switzerland

• Italy

- Another EU country
- Other

Where do you live (postal code)? *

Please enter your answer here:

Pregnancy *

Answer only if the selected gender was 'Female' or 'Diverse'.

- I am currently pregnant
- I have been pregnant once
- I have been pregnant multiple times
- I am currently pregnant and have been pregnant before
- I am NOT pregnant and have NEVER been pregnant

Do you have children? *

Please select only one of the following answers:

- Yes
- No

How many children do you have? *

Answer only if the answer to 'Do you have children?' was 'Yes'.

- 1
- 2
- 3
- More than 3

Health

What does health mean to you? (Rank up to 3 answers) *

All answers must be different. Rank a maximum of 3 answers in order of preference (1 to 8).

• A state of complete mental, physical, and social well-being

• Freedom from illness and ailments

• Feeling good – even without diagnoses or test results

• Not suffering from any diagnosed illness

• Being able to live everyday life without limitations

• Being mentally and physically resilient

• Being happy and free of complaints

• Being exceptionally high-performing

What does health during pregnancy mainly mean to you? *

Please select only one of the following answers:

• A pregnancy without complications

• A pregnancy in which the child develops ideally through provision of all necessary nutrients

• A pregnancy in which the child develops in line with medical developmental expectations

• A pregnancy in which one feels well – even without medical confirmation of health status

• A pregnancy that proceeds as expected and on schedule

Vegan Diet: Definition and Motivation

How do you eat? (Multiple answers possible) *

Select all applicable options

• I ALWAYS follow a strictly plant-based (vegan) diet

• I ALMOST ALWAYS eat vegan, but I occasionally consume honey and/or other bee products (e.g., propolis, royal jelly)

• I ALMOST ALWAYS eat vegan, but I occasionally consume dairy products (e.g., milk, butter, yogurt)

• I ALMOST ALWAYS eat vegan, but I occasionally eat fish and/or seafood (e.g., shrimp, mussels)

• I ALMOST ALWAYS eat vegan, but I occasionally eat meat (e.g., chicken)

• I ALMOST ALWAYS eat vegan, but I occasionally eat fish and meat

How often do you make an exception to your vegan diet? *

Answer only if you selected any of the 'almost always vegan' options above.

• About once or twice a year

• About once or twice every six months

• About once or twice a month

• About once or twice a week

Is a vegan diet healthy? *

Please select only one of the following answers:

• Yes

• No

• Don't know

Why do you eat vegan? (Multiple answers possible) *

Select all applicable options

• For health reasons

• For animal welfare

• For climate protection

• Because I don't like the taste of animal products

• Other

When did you start eating vegan? *

Answer only if you are or have been pregnant.

• Before my pregnancy/pregnancies

• During my pregnancy/pregnancies

• Between two pregnancies (i.e., not during every pregnancy)

In which trimester of pregnancy did you start eating vegan? *

Answer only if you selected 'During my pregnancy/pregnancies' above.

• First trimester

• Second trimester

• Third trimester

Did you follow a strictly vegan diet throughout your (current/most recent) pregnancy?*

Answer only if you are or have been pregnant.

• I continued eating vegan

• I ate vegetarian

• I also ate fish

• I also ate fish and meat

Why did you change your vegan diet? (Multiple answers possible) *

Answer only if you ate vegetarian, fish, or meat during pregnancy.

• Fear of harming my child with a vegan diet

- Because my physician advised me to
- Because my pharmacist advised me to
- Because my dietitian advised me to
- Because my midwife advised me to
- Because friends, family, and/or colleagues advised me to
- Because other vegans advised me to
- Due to information from the internet or social media (Instagram, Facebook, etc.)
- Due to information from scientific literature and journals
- Due to information from magazines, newspapers, and/or lifestyle magazines
- Other

Why did you NOT change your vegan diet? (Multiple answers possible) *

Answer only if you continued eating vegan during pregnancy.

- All nutrients for mother and child were covered
- Because I took supplements to prevent nutrient deficiencies
- Because my physician advised me to continue
- Because my pharmacist advised me to continue
- Because my dietitian advised me to continue
- Because my midwife advised me to continue
- Because friends, family, and/or colleagues advised me to continue
- Because other vegans advised me to continue
- Due to information from the internet or social media (Instagram, Facebook, etc.)
- Due to information from scientific literature and journals
- Due to information from magazines, newspapers, and/or lifestyle magazines
- I did not consider changing my diet

• Other

How long have you been eating vegan? *

• Less than a month

• About 3 months

• About 6 months

• About 1 year

• About 1-3 years

• About 3-5 years

• About 5-10 years

• More than 10 years

Dietary Supplements: Definition and Understanding

Which dietary supplements do you know? Name up to three examples *

Please enter your answer here:

What are dietary supplements? (Multiple answers possible) *

• Foods that supplement the normal human diet

• Concentrates to be taken in small, measured amounts in dosage form

• Typical components of dietary supplements include vitamins, minerals, essential fatty acids, amino acids, and plant extracts

• Dietary supplements are medications

• Dietary supplements are foods like candy, sodas, teas, etc.

• Don't know

How do dietary supplements work? *

Please select only one of the following answers:

• Like prescription medications

• Like over-the-counter medications

• Like food

• Like placebos (inactive substances)

• Cannot be compared to any of the above

• Don't know

Dietary Supplements: Benefits, Risks, and Expectations

How do dietary supplements affect health? *

Please select only one of the following answers:

• Dietary supplements are always beneficial for health

• Dietary supplements can have a positive effect on health

• Dietary supplements have no effect on health

• Dietary supplements can be harmful to health

• Dietary supplements are always harmful to health

• Don't know

Please evaluate the following statements: *

Select the appropriate answer for each item:

Applies, Rather applies, Rather does not apply, Does not apply, Don't know

Taking dietary supplements during times of physical stress (e.g., pregnancy, child development, illness, stress) promotes health

Dietary supplements are particularly beneficial to the health of vegans

Dietary supplements are beneficial to the health of pregnant women

Dietary supplements have a negative effect on the health of the mother and child during pregnancy

Pregnant vegans should take dietary supplements more than non-vegan pregnant women

Dietary Supplements: Personal Selection Criteria

Please answer the following questions with yes or no: *

Do you take dietary supplements?

• Yes

• No

Have you ever received advice on dietary supplements?

• Yes

• No

Should physicians, pharmacists, and dietitians provide more guidance on dietary supplements for pregnant vegans?

• Yes

• No

Do you support the use of dietary supplements?

• Yes

• No

Do you support the use of dietary supplements (e.g., multivitamins, combination products, individual nutrients) during pregnancy?

• Yes

• No

What are your main reasons for taking dietary supplements? (Rank up to 5 answers) *

Answer only if you answered 'yes' to taking supplements. Please rank up to 5 different answers.

• Dietary supplements support health

• Essential nutrients lacking in a vegan diet can be supplemented

• My physician advised me to take supplements

• My pharmacist advised me to take supplements

• My dietitian advised me to take supplements

• My friends, family, or colleagues advised me to take supplements

• Motivated by the internet and social media (Instagram, Facebook, etc.)

• Motivated by advertising

• Motivated by scientific literature and journals

• Motivated by magazines, newspapers, and/or lifestyle magazines

• Other

Why do you reject dietary supplements during pregnancy? (Multiple answers possible) *

Only answer this question if you previously stated that you do not support the use of dietary supplements during pregnancy.

• Supplements are poorly absorbed by the body

• All essential nutrients can be obtained from a vegan diet

• Supplements harm the health of the pregnant person

• Supplements harm the health of the unborn child

• Uncertain if supplements are vegan

• Physician advised against taking them

• Pharmacist advised against taking them

- Dietitian advised against taking them
- Friends, family, or colleagues advised against taking them
- Other

Why do you support the use of dietary supplements during pregnancy? (Multiple answers possible) *

Only answer this question if you previously stated that you support the use of dietary supplements during pregnancy.

- Supplements support the positive development of the child
- Supplements support the health of the pregnant person
- Essential nutrients missing from the vegan diet can be supplemented
- Supplements meet the increased nutritional needs during pregnancy
- Physician advised taking supplements
- Pharmacist advised taking supplements
- Dietitian advised taking supplements
- Friends, family, or colleagues advised taking supplements
- Other

Do you make sure your supplements are vegan? *

Only answer this question if you previously stated that you support the use of dietary supplements.

- Yes
- No
- I don't take dietary supplements

Which dietary supplements do you take? (Multiple answers possible) *

Only answer this question if you previously answered ‘yes’ to supporting the use of dietary supplements.

• Vitamin B12

• Folic acid

• Omega-3 fatty acids (DHA/EPA)

• Iron

• Vitamin D

• Iodine

• Zinc

• Calcium

• Magnesium

• Combination products (e.g., multivitamins)

• None

• Other

Which dietary supplements did you take or are you taking during your pregnancy/pregnancies? (Multiple answers possible) *

Only answer this question if you identified as female or diverse, and are currently pregnant or have experienced one or more pregnancies.

• Vitamin B12

• Folic acid

• Omega-3 fatty acids (DHA/EPA)

• Iron

• Vitamin D

• Iodine

• Zinc

• Calcium

• Magnesium

• Combination products (e.g., prenatal multivitamins)

• None

• Other

Dietary Supplements: Knowledge Level

Please answer the following questions by selecting the appropriate response: *

Options: Applies / Rather applies / Rather does not apply / Does not apply / Don't know

• I am well informed about dietary supplements for pregnant vegans

Which dietary supplements can pregnant vegans safely take without consulting a physician, pharmacist, and/or dietitian? (Multiple answers possible) *

• Vitamin B12

• Folic acid

• Omega-3 fatty acids (DHA/EPA)

• Iron

• Vitamin D

• Iodine

• Zinc

• Calcium

• Magnesium

• Combination products (e.g., multivitamins)

• None

• Other

Which dietary supplements were recommended to you during your pregnancy/pregnancies by professionals (physician, pharmacist, and/or dietitian)? (Multiple answers possible) *

Only answer this question if you identified as diverse or female, and are currently pregnant or have experienced one or more pregnancies.

• Vitamin B12

• Folic acid

• Omega-3 fatty acids (DHA/EPA)

• Iron

• Vitamin D

• Iodine

• Zinc

• Calcium

• Magnesium

• Combination products (e.g., multivitamins)

• None

• Other

• Advice was not received

Can pregnant vegans meet their nutrient needs through a purely plant-based diet? *

• Yes

• No

• Don't know

Can potential nutrient deficiencies in a vegan pregnancy be compensated by supplements? *

- Yes
- No
- Don't know

Dietary Supplements: Information Sources

Where do you get your information on supplement use during pregnancy? (Multiple answers possible) *

- Friends, family, colleagues
- General practitioner
- Pediatrician
- Gynecologist
- Pharmacist
- Dietitian
- Midwife
- Other vegans
- Physiotherapist
- Fitness trainer
- Vegan organizations
- Scientific literature/journals
- Internet and social media (Instagram, Facebook etc.)
- Newspapers, magazines and/or lifestyle magazines
- I do not seek information about supplements in pregnancy
- Advertising
- Other

Where is the best place to get advice on dietary supplements during pregnancy? (Rank up to 3 answers) *

• Friends, family, colleagues

• General practitioner

• Pediatrician

• Gynecologist

• Pharmacist

• Dietitian

• Midwife

• Other vegans

• Physiotherapist

• Fitness trainer

• Vegan organizations

• Scientific literature/journals

• Internet and social media (Instagram, Facebook etc.)

• Newspapers, magazines and/or lifestyle magazines

• I do not seek information about supplements in pregnancy

• Advertising

• Other

How would you describe the information you have received about dietary supplements? *

• Trustworthy

• Helpful

• Unclear

• Not very informative

- Insufficient
- I do not receive information about supplements

Is it easy to access information on dietary supplements for pregnant vegans? *

- Yes
- No
- Don't know

How do you evaluate the available advice/information on dosage of dietary supplements during pregnancy? *

- Very good
- Good
- Adequate
- Inadequate
- Don't know

Vegan Diet: Influencing Factors

What caused you the most uncertainty about maintaining a vegan diet during your pregnancy/pregnancies? (Multiple answers possible, max 3) *

Only answer this question if you identified as diverse or female, and are currently pregnant or have been pregnant once or multiple times.

- Social environment (family, friends, colleagues)
- Treating physicians
- Advice from pharmacist
- Dietitian
- Midwife

• Internet and social media

• Scientific literature/journals

• Newspapers, magazines, and/or lifestyle magazines

• General lack of trustworthy information on vegan diet and supplements during pregnancy

• I was not uncertain

• Other

Did you inform your gynecologist during pregnancy that you follow a vegan diet? *

Only answer this question if you identified as female or diverse, and are currently pregnant or have had one or more previous pregnancies.

• Yes

• No

Did your gynecologist advise you on dietary supplements during pregnancy? *

Only answer this question if you identified as female or diverse, and are currently pregnant or have experienced one or more pregnancies in the past.

• Yes, I received comprehensive advice

• I received minimal advice

• I did not want to be advised

• No, I received no advice

Dietary Supplements: Deficiency and Overdosage Risks

Please assess the health risks of nutrient deficiency (underdosing) and overdosage due to a vegan diet during pregnancy. Use the following scale for each item:

1 = No Risk, 5 = High Risk, 'Don't know' = If you are unsure.

Risk Assessment ***– vegan pregnancy***

For each nutrient listed, please indicate:

- The perceived risk if there is a deficiency or underdosage.

- The perceived risk if there is an overdosage.

- Vitamin B12:

Risk of Deficiency/Underdosing: 1 2 3 4 5 Don't know

Risk of Overdosage: 1 2 3 4 5 Don't know

- Folic Acid:

Risk of Deficiency/Underdosing: 1 2 3 4 5 Don't know

Risk of Overdosage: 1 2 3 4 5 Don't know

- Omega-3 Fatty Acids (DHA/EPA):

Risk of Deficiency/Underdosing: 1 2 3 4 5 Don't know

Risk of Overdosage: 1 2 3 4 5 Don't know

- Iron:

Risk of Deficiency/Underdosing: 1 2 3 4 5 Don't know

Risk of Overdosage: 1 2 3 4 5 Don't know

- Vitamin D:

Risk of Deficiency/Underdosing: 1 2 3 4 5 Don't know

Risk of Overdosage: 1 2 3 4 5 Don't know

- Iodine:

Risk of Deficiency/Underdosing: 1 2 3 4 5 Don't know

Risk of Overdosage: 1 2 3 4 5 Don't know

- Zinc:

Risk of Deficiency/Underdosing: 1 2 3 4 5 Don't know

Risk of Overdosage: 1 2 3 4 5 Don't know

- Calcium:

Risk of Deficiency/Underdosing: 1 2 3 4 5 Don't know

Risk of Overdosage: 1 2 3 4 5 Don't know

- Magnesium:

Risk of Deficiency/Underdosing: 1 2 3 4 5 Don't know

Risk of Overdosage: 1 2 3 4 5 Don't know

Dietary Supplements: Administration and Compliance

Main concern during supplement intake in pregnancy

Only answer this question if you are female or diverse and are currently pregnant or have been pregnant at least once before. Please select only one of the following answers:

• My unborn child could be harmed due to incorrect dosage

• My unborn child and I could be negatively affected by the intake of supplements

• My body is more burdened than supported by the supplement

• The health of my unborn child could be negatively affected by taking supplements (even with correct dosage)

• I have no concerns as supplements are safe

• Other

Supplement use in pregnancy

Please select the appropriate answer for each statement:

• I take/took supplements during pregnancy: Always / Mostly / Rarely / Never / No answer

Please rate the following statements

• Regular intake of supplements during pregnancy is important for their effectiveness: Yes / Rather Yes / Rather No / No / Don’t know

• Clear dosage recommendations are a prerequisite for me to take supplements during pregnancy as a vegan: Yes / Rather Yes / Rather No / No / Don’t know
